# Time-Course Transcriptome Analysis Reveals Distinct Transcriptional Regulatory Networks in Resistant and Susceptible Grapevine Genotypes in Response to White Rot

**DOI:** 10.3390/ijms252111536

**Published:** 2024-10-27

**Authors:** Tinggang Li, Xing Han, Lifang Yuan, Xiangtian Yin, Xilong Jiang, Yanfeng Wei, Qibao Liu

**Affiliations:** Shandong Academy of Grape, Shandong Academy of Agricultural Sciences, No. 1-27, Shanda South Road, Jinan 250100, China; litinggang@saas.ac.cn (T.L.); hanxing@nwafu.edu.cn (X.H.); yuanlifang@saas.ac.cn (L.Y.); yinxiangtian@saas.ac.cn (X.Y.); jiangxilong@saas.ac.cn (X.J.); weiyanfeng@saas.ac.cn (Y.W.)

**Keywords:** white rot, *V. vinifera*, TO-GCN, JA, disease resistance

## Abstract

Grapevine (*Vitis vinifera* L.) is a globally significant economic crop. However, its widely cultivated varieties are highly susceptible to white rot disease. To elucidate the mechanisms of resistance in grapevine against this disease, we utilized time-ordered gene co-expression network (TO-GCN) analysis to investigate the molecular responses in the grapevine varieties ‘Guifeimeigui’ (GF) and ‘Red Globe’ (RG). An assessment of their resistance demonstrated that GF is highly resistant to white rot, whereas RG is highly susceptible. We conducted transcriptome sequencing and a TO-GCN analysis on leaf samples from GF and RG at seven time points post-infection. Although a significant portion of the differentially expressed genes related to disease resistance were shared between GF and RG, the GF variety rapidly activated its defense mechanisms through the regulation of transcription factors during the early stages of infection. Notably, the gene *VvLOX3*, which is a key enzyme in the jasmonic acid biosynthetic pathway, was significantly upregulated in GF. Its upstream regulator, *Vitvi08g01752*, encoding a HD-ZIP family transcription factor, was identified through TO-GCN and yeast one-hybrid analyses. This study provides new molecular insights into the mechanisms of grapevine disease resistance and offers a foundation for breeding strategies aimed at enhancing resistance.

## 1. Introduction

Grapevine (*V. vinifera* L.) is a significant crop that is highly valuable throughout the world. However, its widely cultivated varieties are highly susceptible to various fungal pathogens, particularly white rot caused by *Coniella vitis* [1]. This fungal pathogen poses a severe threat to the growth of grapevines and the production of grapes by infecting injured grapes, rachises, young shoots, and leaves, particularly under warm and humid conditions, and can lead to annual losses of 10–20% in yields [2]. Antifungal chemical agents are commonly used to control white rot. However, the prolonged use of these fungicides has resulted in increased resistance to pathogens and concerns about food safety and environmental pollution. Therefore, a more thorough understanding of the pathogenic mechanisms of white rot and its impact on grapevines is crucial to develop more sustainable disease control strategies and breed resistant varieties.

Plant immunity is a complex defense system that plants rely on to recognize and effectively respond to pathogen invasion. This immune mechanism primarily operates on two levels: pattern-triggered immunity (PTI) and effector-triggered immunity (ETI). Plant hormones play a critical role in regulating PTI and ETI [3]. Salicylic acid (SA) is typically associated with defense against biotrophic pathogens, while jasmonic acid (JA) and ethylene (ET) are more involved in resistance to necrotrophic pathogens [4,5]. For example, SA enhances resistance by promoting the expression of defense genes through the activation of the non-expresser of PR genes 1 (NPR1)-dependent pathway [6]. NPR1 directly binds to salicylic acid (SA), resulting in a conformational change in NRR1, which subsequently facilitates transcriptional regulation through its interaction with TGA-type transcription factors [7]. These activated TGA transcription factors promote the expression of pathogenesis-related (PR) genes, which, in turn, triggers the plant immune response [8]. Conversely, JA activates genes that are related to defense by interacting with the coronatine insensitive 1 (COI1) receptor [9]. The jasmonoyl–isoleucine (JA–Ile) conjugate promotes the binding of SCF^COI1^ to the jasmonate ZIM-domain 1 (JAZ1) repressor protein, leading to the subsequent degradation of the JAZ1 protein [10]. This degradation then allows transcription factors such as the basic helix–loop–helix transcription factor MYC2 to activate JA response genes [11]. Additionally, the antagonistic and cooperative interactions between JA and SA suggest that plants prioritize the most beneficial defense pathway under limited resources [12]. Therefore, an in-depth investigation of the regulatory networks that utilize plant hormones in immunity is highly significant to improve plant disease resistance.

To date, several studies have reported the molecular mechanisms that underlie the resistance of grapevine to white rot, which has revealed the key resistance genes and their associated metabolic pathways. Genome-wide association studies (GWASs) have shown that populations of wild Chinese grape are highly resistant to white rot and are highly diverse genetically [13]. The *VvWRKY5* gene significantly enhances the resistance of grapevine to white rot by activating the JA signaling pathway [14]. The *VaRPP13* gene, which acts through the SA signaling pathway, regulates the expression of downstream defense genes, which further enhances the degree of resistance [15]. Moreover, studies that integrated metabolomics and transcriptomics have demonstrated the critical role of the flavonoid metabolic pathways in the resistance of grapevine to white rot. This is particularly true in *V. davidii*, in which the significant activation of this pathway may inhibit the invasion of pathogens by enhancing the activity of antioxidants and cell wall strength [16]. Despite these advances, the molecular mechanisms of grapevine resistance to white rot remain unclear.

The use of TO-GCN was initially proposed to analyze time-series transcriptome data with the goal of revealing the dynamic changes in gene expression and their transitions during biological processes [17]. This method overcomes the limitations of traditional co-expression network analysis, which cannot analyze temporal dynamics. This makes it particularly suitable to study gene regulatory mechanisms under different developmental stages and environmental conditions [17]. In subsequent studies, TO-GCNs have been widely applied across various species and research contexts. For example, a TO-GCN identified key regulatory modules associated with the Ca^2+^ signaling pathways in the response of sugarcane (*Saccharum officinarum* L.) to sugarcane smut (*Sporisorium scitamineum*) [18]. Additionally, a TO-GCN has been used to study the recurrent release of aromatic compounds in *Rosa yangii* [19], flavonoid metabolism during the development of leaves in *Epimedium* sp. [20], and the response of *Pinus tabuliformis* Carr. to UV radiation [21]. This technique successfully identified critical regulatory networks and gene modules. Thus, TO-GCNs have shown great potential at uncovering gene regulatory mechanisms under temporal dynamics.

In this study, time-course transcriptome sequencing at seven time points was utilized to explore the molecular mechanisms that underlie the differential resistance to white rot in the grapevine varieties GF and RG. The construction of TO-GCNs enabled this study to elucidate the temporal differences in the responses of the GF and RG varieties to disease. The GF variety rapidly activated more transcription factors (TFs) related to disease resistance and the resistance genes that were activated during the early stages of pathogen infection. In particular, there was a significant increase in the levels of expression of key enzymes and regulatory factors in the biosynthetic and signaling pathways of hormones in plants. We observed that the expression of the key enzyme VvLOX3 in the JA biosynthetic pathway was significantly enhanced and closely associated with resistance in GF. The combination of the TO-GCN with the yeast one-hybrid (Y1H) technique enabled the identification of *Vitvi08g01752*, encoding a HD-ZIP family transcription factor, as an upstream TF of *VvLOX3*, which may enhance the resistance of GF by regulating *VvLOX3*. Based on these findings, a model of the regulation of JA biosynthesis during infection with white rot pathogen was proposed, and it reveals the molecular mechanisms of the resistance to white rot in GF. The results of this study not only provide new perspectives to further elucidate the mechanisms of grapevine resistance to white rot but also lay a theoretical foundation for the molecular breeding of resistant varieties.

## 2. Results

### 2.1. Phenotypic Responses of GF and RG to C. vitis Inoculation

**Figure 1:**
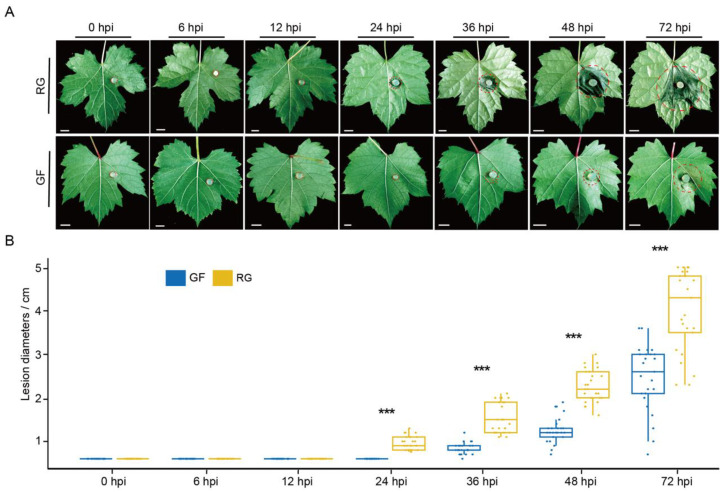
Resistance assessment of GF and RG varieties defense against *C. vitis*. (**A**,**B**) show the phenotypes and lesion diameters of GF and RG varieties following inoculation with *C. vitis* at 0, 6, 12, 24, 36, 48, and 72 hpi. The red dashed line is used to mark the boundaries of the diseased area on the leaves. Bar = 1 cm. For each cultivar, the lesion diameters of 30 leaves were counted. Significant differences are indicated by different letters (*** *p* < 0.001).

To evaluate the resistance of the GF and RG varieties to white rot, detached leaves of similarly growing GF and RG plants were inoculated with disks of *C. vitis*, and the symptoms of disease were observed at seven time points, including 0, 6, 12, 24, 36, 48, and 72 hpi. Interestingly, visible lesions appeared on the RG leaves at 24 hpi, whereas no lesions were observed on the GF leaves until 36 hpi (Figure 1A). As the infection progressed, there were significantly larger diameters of the lesions on the RG leaves, while the RG leaves exhibited more severe symptoms of decay (Figure 1B). By 72 hpi, the lesions on the RG leaves covered almost one-third of the total leaf area, with an average lesion diameter of 4.02 cm compared to only 2.46 cm on the GF leaves. These results indicate that the GF is indeed more resistant to white rot than RG.

### 2.2. Transcriptome Sequencing Analysis

A total of 42 RNA samples (21 samples from each variety) were sequenced and generated an average of 70.77 million raw reads per sample. These ranged from 52.81 to 95.68 million. After quality control, the clean reads were aligned to the PN40024 12X.v2 reference genome, and the unique mapping rates ranged from 81.48% to 91.70% (Table S1). A total of 16,461 expressed genes (FPKM ≥ 1; at least three samples) were detected, and 16,208 (98.45% of all the genes expressed) were shared between GF and RG (Figure S1A). This indicates that mostly similar categories of genes were expressed between the two varieties during infection by *C. vitis*. DESeq2 was used to identify the differentially expressed genes (DEGs) between the non-inoculated (0 hpi) and inoculated (6, 12, 24, 36, 48, and 72 hpi) samples from GF and RG (Table S2). Unlike the expressed genes, there were 3087 DEGs (47.31%) shared between GF and RG, while 1640 DEGs (25.13%) were specific to GF, and 1798 DEGs (27.56%) were specific to RG (Figure S1B). This suggests that while GF and RG share common transcriptional regulatory mechanisms in response to infection with *C. vitis*, they also possess species-specific regulatory mechanisms.

**Figure 2:**
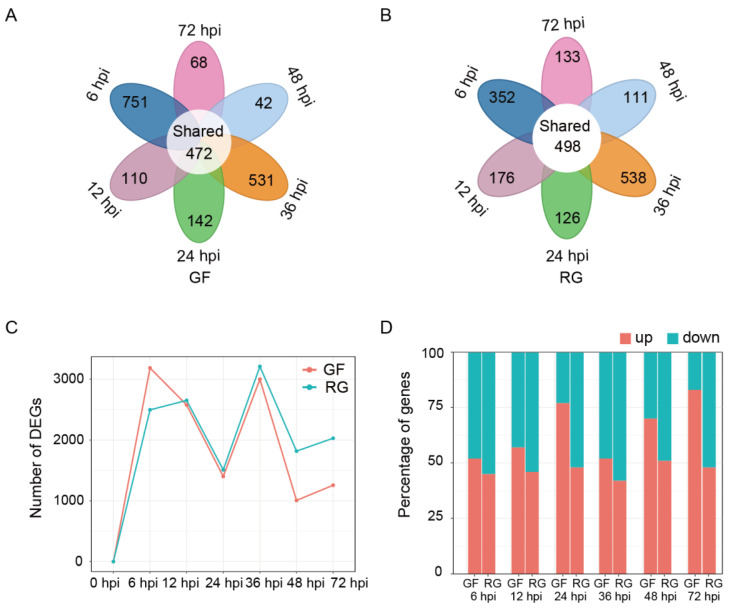
Expression patterns of GF and RG varieties after inoculation with *C. vitis*. (**A**,**B**) Shared and unique DEGs across various time points after inoculation with *C. vitis* in GF and RG varieties. (**C**) The number of DEGs at various time points. (**D**) The percentage of upregulated and downregulated DEGs at various time points.

In the GF variety, compared to the 0 hpi sample, 3185, 2577, 1400, 3000, 1006, and 1256 DEGs were detected in the 6, 12, 24, 36, 48, and 72 hpi samples, respectively, with 472 DEGs shared across all six time points (Figure 2A). Similarly, in the RG variety, compared to the 0 hpi sample, 2496, 2651, 1509, 3210, 1817, and 2029 DEGs were detected in the 6, 12, 24, 36, 48, and 72 hpi samples, respectively; 498 DEGs were shared across all six time points (Figure 2B). Both GF and RG exhibited two peaks of transcriptional upregulation in response to infection with *C. vitis*. They occurred at 6 hpi and 36 hpi (Figure 2C). Interestingly, compared to RG, GF had more upregulated DEGs than downregulated ones at all time points, and there was a significant increase in the proportion of upregulated genes following infection with *C. vitis* (Figure 2D).

### 2.3. Functional Analysis of the DEGs

**Figure 3:**
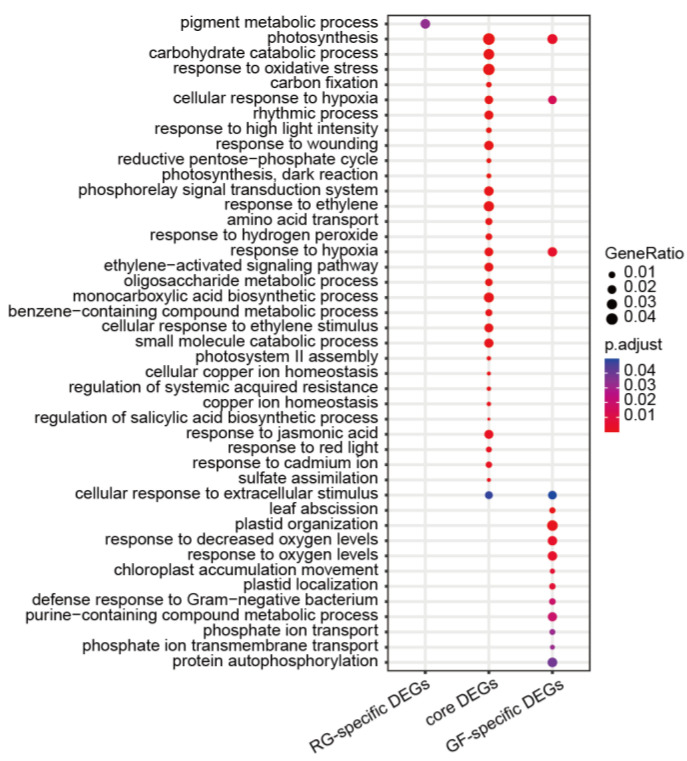
GO analysis of RG-specific DEGs, core DEGs, and GF-specific DEGs. The shared DEGs between GF and RG were categorized as core DEGs, while the unique DEGs were classified into RG-specific and GF-specific DEGs.

To further investigate the molecular mechanisms that underlie the resistance to white rot in grape, the DEGs in GF and RG were categorized into three groups. These included RG-specific DEGs (1796), core DEGs (3087), and GF-specific DEGs (1640) (Table S3). These genes were then subjected to functional annotation and enrichment analysis (Figure 3). Both the RG-specific and GF-specific DEGs were enriched in relatively few gene ontology (GO) terms. The RG-specific DEGs were significantly enriched in only one GO term, pigment metabolic process. In contrast, the GF-specific DEGs were enriched in 15 significant GO terms, and only protein autophosphorylation was potentially related to disease resistance. Interestingly, the core DEGs were significantly enriched in several GO terms related to plant defense, such as response to ethylene, the ethylene-activated signaling pathway, regulation of systemic acquired resistance, response to jasmonic acid, and regulation of the salicylic acid biosynthetic process.

### 2.4. TO-GCN of GF and RG Under C. vitis Inoculation

**Figure 4:**
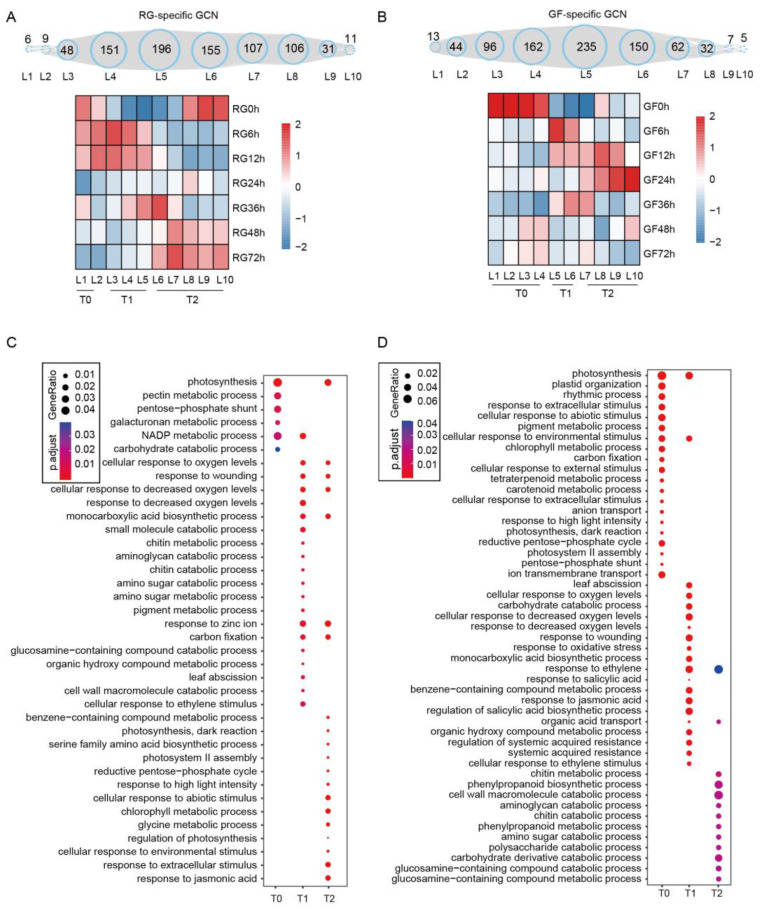
Construction of TO-GCNs and the functions of co-expression genes. (**A**,**B**) The TO-GCNs were constructed with TF genes for RG and GF varieties. In a TO-GCN, each node (blue dotted circles) represents a TF gene and the number in each level represents the number of TF genes at that level (Top). The heatmaps were generated with the mean z-score of TF gene expression at each level (Bottom). T0: the uninoculated stage; T1: the early inoculation stage; T2: the late inoculation stage. (**C**,**D**) GO enrichment analysis for T0, T1, and T2 group genes in the RG-specific GCN and GF-specific GCN.

The transcriptome dynamics of the DEGs revealed differences between the two varieties of grapevine in response to white rot infection. However, the primary differences in the transcriptional reprogramming along the infection timeline remain unclear. To address the complexity of the time-series data, we utilized TO-GCN to analyze our transcriptome data. The FPKM values of 834 expressed TFs were used to calculate Pearson correlation coefficients (PCCs) and construct time-ordered GCNs specific to GF (PCC ≥ 0.85 for GF and −0.5 ≤ PCC < 0.5 for RG). Similarly, GCNs specific to RG were constructed (PCC ≥ 0.85 for RG and −0.5 ≤ PCC < 0.5 for GF). These specified levels of TO-GCN corresponded to the time-series of gene expression in GF and RG following infection with *C. vitis*. A total of 820 expressed TF genes formed 10 time-ordered levels in the RG-specific GCN (Figure 4A and Table S4), which revealed the timeline of the transcriptional response of RG to infection with *C. vitis*. Levels L4–L8 contained the most TF genes, with 151, 196, 155, 107, and 106 genes, respectively. Based on their patterns of expression, the time-ordered subnetworks were further divided into different stages, including the uninoculated stage (T0; 0 hpi; corresponding to L1–L2), the early inoculation stage (T1; *C. vitis* infection at 6–12 hpi; corresponding to L3–L5), and the late inoculation stage (T2; *C. vitis* infection at 24–72 hpi; corresponding to L6–L10) (Figure 4A). Most of the TF genes appeared in the T1 and T2 periods, with 395 and 410 TF genes, respectively. For the GF-specific GCN, 806 TF genes formed 10 time-ordered levels (Figure 4B and Table S5). Among these, L4, L5, and L6 contained the most TF genes, with 162, 235, and 150 TF genes, respectively. Heatmaps of TF gene expression patterns indicated that TF genes in L5 and L6 were upregulated at 6 hpi, suggesting that L5 and L6 were the earliest responders to *C. vitis* infection in GF and also participated in stress responses at 12 hpi and 36 hpi. Subsequently, TF genes in L7, L8, and L9 were upregulated starting at 12 hpi. Similarly, for the GF-specific GCN, T0 corresponded to levels L1–L4; T1 corresponded to levels L5–L6; and T2 corresponded to levels L7–L10 (Figure 4B). Unlike the RG-specific GCN, in the GF-specific GCN, T1 and T2 contained more TF genes with 312 and 385 genes, respectively. These results suggest that GF and RG respond at different transcriptional regulatory speeds in response to infection with *C. vitis*.

To further study the biological functions of the structural genes regulated by the TFs in RG and GF during the response to white rot infection, the co-expression relationships between the TFs and non-TFs (only DEGs) were defined using similar thresholds (i.e., PCC ≥ 0.85). Subsequently, the TF and non-TF genes were annotated and subjected to functional enrichment at the different stages of infection. In the RG-specific GCN, the DEGs associated with T0 (the uninoculated stage) were primarily enriched in terms related to plant growth and development, such as photosynthesis and carbohydrate catabolic process. The DEGs associated with T1 (the early inoculation stage) were enriched in multiple GO terms, and a few were related to plant defense, such as response to wounding, chitin metabolic process, and cellular response to ethylene stimulus. In contrast, the DEGs associated with T2 (the late inoculation stage) were enriched in only one GO term related to plant immunity, which was response to JA (Figure 4C). As expected, the enrichment results of the GF-specific GCN differed significantly from those of the RG-specific GCN, particularly in defense-related GO terms (Figure 4D). Most notably, in the GF-specific GCN, the DEGs associated with T2 were enriched in multiple plant responses to biotic stress-related terms, including response to wounding, response to oxidative stress, response to ethylene, response to salicylic acid, response to jasmonic acid, regulation of the salicylic acid biosynthetic process, and systemic acquired resistance. Additionally, the DEGs associated with T2 were primarily enriched in the metabolism and catabolism of plant cell wall components, such as amino polysaccharides, chitin, polysaccharides, and carbohydrates. These results suggest that the DEGs from GF contain more genes related to plant immunity, which are highly responsive during the early stages of white rot infection (T1). Thus, they confer stronger resistance to the white rot pathogen compared to RG.

### 2.5. Responses of Plant Hormone-Related Genes to C. vitis Inoculation in GF and RG

**Figure 5:**
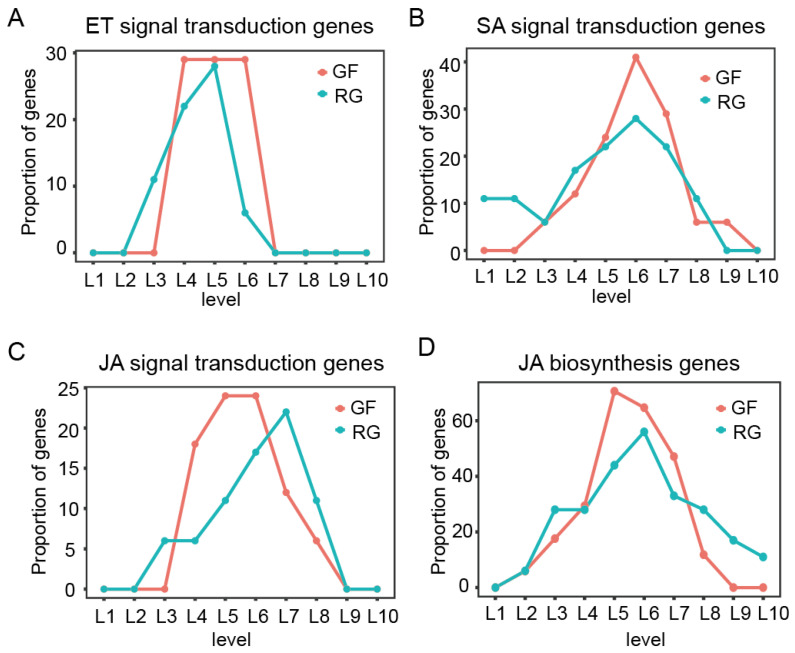
The proportions of plant hormone-related genes at each level in GF-specific and RG-specific GCNs. (**A**–**C**) Genes in ET, SA, and JA hormone signal transduction pathways. (**D**) Genes in JA hormone biosynthesis pathway. In RG, levels L1 and L2 corresponded to the T0 stage (0 hpi), levels L3 to L5 corresponded to the T1 stage (6–12 hpi), and levels L6 to L10 corresponded to the T2 stage (24–72 hpi). In GF, levels L1 to L4 corresponded to the T0 stage (0 hpi), levels L5 and L6 corresponded to the T1 stage (6 hpi), and levels L7 to L10 corresponded to the T2 stage (12–72 hpi).

Plant hormones play a crucial role in plant immunity. The results from the TO-GCN analysis indicated that GF produced plant hormones earlier and more rapidly than RG, thereby mediating its resistance to white rot (Figure 4C,D). Consequently, the proportion of genes in the three hormone signaling pathways (ET, SA, and JA) at each level of TO-GCN was analyzed in more detail (Table S6). In GF and RG, 17 and 18 DEGs, respectively, were annotated to the ko040745 (plant hormone signal transduction) pathway (Figure 5A). We found that the genes related to the ET signaling pathway appeared during the T1 stage, aligning with the levels L3–L5 in RG and levels L5 and L6 in GF, with some also appearing at L4. Then, these genes gradually diminished by the T2 stage (Figure 5B). There were similar trends in the proportion of genes related to the SA signaling pathway in both the GF and RG varieties, with the number of genes increasing during the T1 stage and then decreasing during the T2 stage. In contrast, the genes related to the JA signaling pathway peaked at L5 and L6 (T1 stage, corresponding to 6 hpi) in GF, whereas they peaked at L7 (T2 stage, corresponding to 72 hpi) in RG (Figure 5C). This suggests the critical role of the JA signaling pathway in grapevine resistance to white rot is consistent with the findings of previous studies. Nevertheless, there has been little research focused on the differences in the biosynthetic pathways of hormones in the resistant and susceptible varieties. Therefore, we also analyzed the proportion of genes in the ET, SA, and JA biosynthetic pathways at each level of TO-GCN. No DEGs related to SA biosynthesis were found, and few DEGs related to ET biosynthesis were identified. Thus, this study focused on the 19 genes related to JA biosynthesis (Table S6). The proportion of these genes in GF increased significantly during the T1 stage (L5 and L6) and peaked at L5 (corresponding to 6 hpi). However, they peaked during the T2 stage in RG (L6, corresponding to 36 hpi) (Figure 5D). Additionally, a much higher proportion of the genes associated with the biosynthesis of JA were at the peak stage in GF than in RG at 70.6% and 56%, respectively. These results suggest that GF can produce JA more quickly than RG in response to white rot infection, which may be a key factor in the strong immunity of GF.

### 2.6. JA Biosynthesis Under C. vitis Inoculation

**Figure 6:**
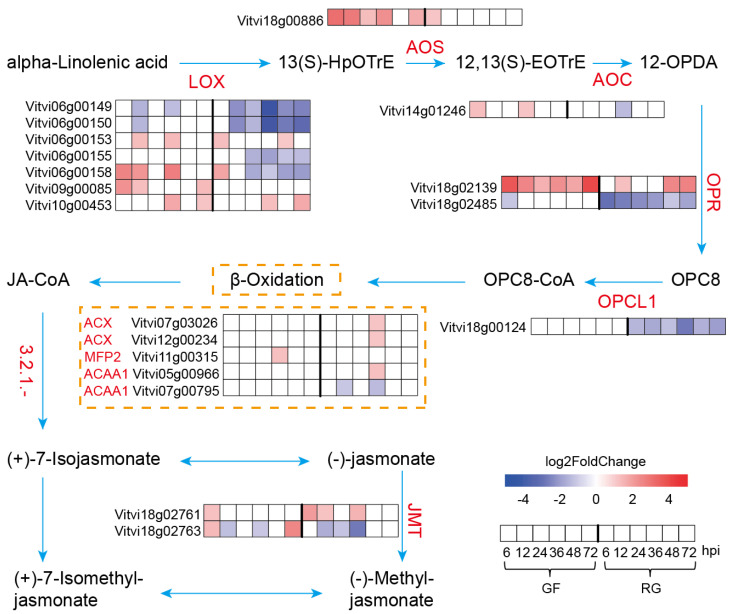
The module of the JA biosynthesis pathway in GF and RG varieties in response to *C. vitis* infection. LOX: lipoxygenase; AOS: hydroperoxide dehydratase; AOC: allene oxide cyclase; OPR: 12-oxophytodienoic acid reductase; OPCL1: OPC-8:0 CoA ligase 1; ACX: acyl-CoA oxidase; MFP2: enoyl-CoA hydratase/3-hydroxyacyl-CoA dehydrogenase; ACAA1: acetyl-CoA acyltransferase 1; and JMT: jasmonate O-methyltransferase. Gene expression profiles at various time points are displayed in the heatmap alongside the names of enzymatic genes.

Accordingly, we proposed a model that summarized the temporal expression of the genes for the JA biosynthesis pathway in the two varieties of grapevine (Figure 6). The JA biosynthetic pathway begins with alpha-linolenic acid and proceeds through several key steps, including the oxidation of alpha-linolenic acid, formation and cyclization of allene oxide, reduction in OPDA, and final β-oxidation. This pathway ultimately produces jasmonic acid and methyl jasmonate. Nine enzymes in the JA biosynthetic pathway, including LOX, AOS, AOC, OPR, OPCL1, ACX, MFP2, ACAA1, and JMT, were expressed at different patterns of temporal expression in the two varieties of grapevine. The key enzymes LOX, AOS, AOC, and OPR were highly expressed in GF following white rot infection, whereas their expression remained unchanged or significantly decreased in RG. Notably, there are nine LOX genes that are homologous to Arabidopsis *AT1G17420* (*AtLOX3*), and six are present as tandem gene duplications on chromosome 6. This suggests that they are important for survival. We focused on one LOX gene, *Vitvi06g00158* (named *VvLOX3*), which exhibited much higher levels of expression (FPKM value) than the other homologous genes. In addition, it was highly expressed in GF but poorly expressed in RG in response to infection with the white rot pathogen.

### 2.7. Upstream Regulators of VvLOX3

**Figure 7:**
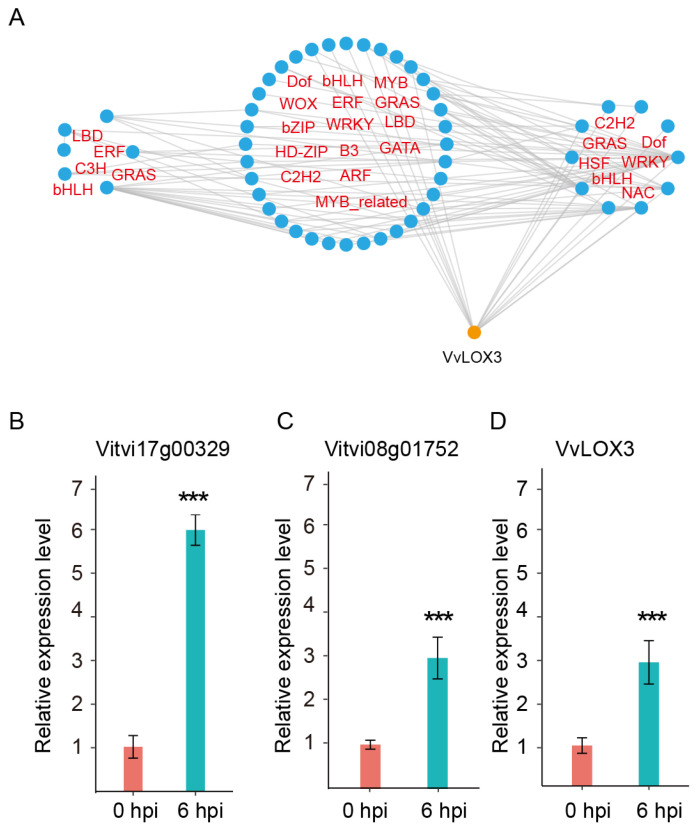
Regulators of the *VvLOX3* gene in grapevine resistance to *C. vitis.* (**A**) Resolved hierarchical regulation for *VvLOX3* in GF-specific GCN. (**B**–**D**) Relative expressions of *Vitvi17g00329*, *Vitvi08g01752*, and *VvLOX3* in GF leaves at 0 and 6 h post-inoculation (hpi) with *C. vitis*. Relative expression levels of three genes were assessed by RT-qPCR. Error bars represent standard errors of three biological replicates; *** *p* < 0.001, according to unpaired Student’s *t*-tests.

Given the significant upregulation of the expression of *VvLOX3* in GF following white rot infection, its upstream regulators are of particular interest. Therefore, we utilized the GF-specific TF GCN to identify upstream regulators of *VvLOX3*, which are associated with the TFs co-expressed at the L5 and L6 levels. First, we used this method to predict direct regulators and indirect TFs for the *VvLOX3*. We then predicted the transcription factor binding sites (TFBSs) in the promoter regions of each *VvLOX3* and TF. For each TF–candidate target gene pair, if the TFBS appeared in the promoter region of the candidate target gene, and the TF was differentially expressed in GF, the TF–candidate target gene pair was retained. Finally, we obtained a more refined subnetwork for *VvLOX3* (Figure 7A and Table S7). We predicted that there were 24 TFBSs in the 2000 kb upstream promoter region of *VvLOX3* and identified 10 and 8 direct regulators (first-order regulators) of *VvLOX3* at the L6 and L5 levels, respectively. These first-order regulators included WRKY (four genes), bHLH (two genes), NAC (two genes), Dof (two genes), C2H2 (one gene), ERF (one gene), GRAS (one gene), HD-ZIP (one gene), HSF (one gene), MYB-related (one gene), and bZIP (one gene) family members (Table S8). Notably, the high proportion of WRKY family members, including *WRKY6* (*Vitvi12g00388*), *WRKY7* (*Vitvi07g01694*), *WRKY22* (*Vitvi07g00421*), *WRKY47* (*Vitvi07g00523*), and *WRKY70* (*Vitvi13g01916*), suggests their potential role in responding to pathogen infection, thereby regulating JA biosynthesis and enhancing the resistance of GF to white rot.

**Figure 8:**
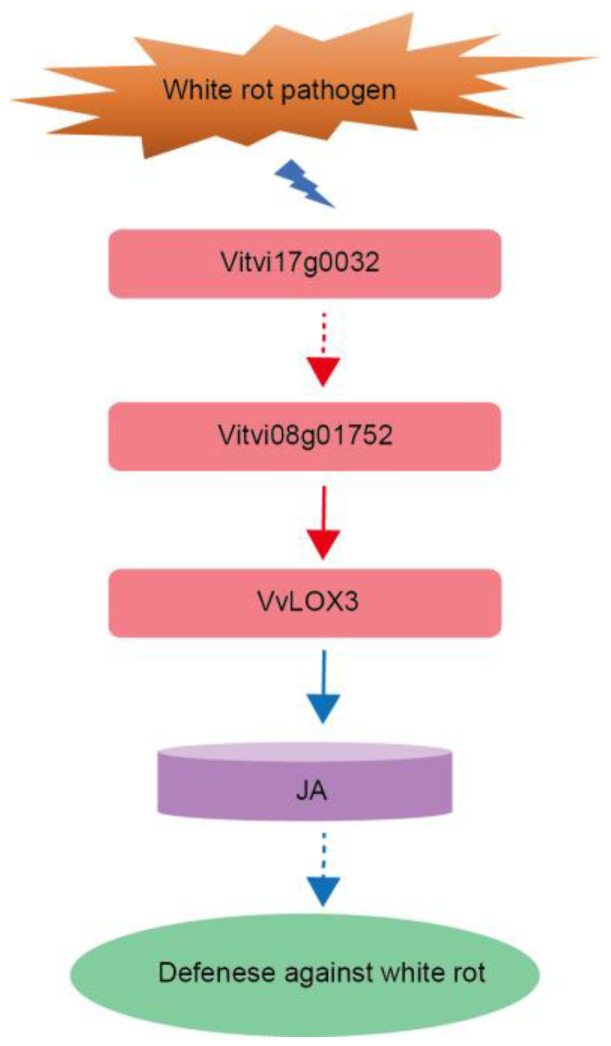
Regulatory model for *VvLOX3* gene in GF response to white rot. Red dashed line with an arrow: validation by qRT-PCR. Red solid line with an arrow: validation by qRT-PCR and Y1H assays.

To further confirm the regulation of *VvLOX3* by the TFs predicted, the promoter region of *VvLOX3* (1998 bp upstream of the CDS) was cloned and inserted into the pAbAi vector, and a Y1H assay was conducted. SD/-Leu medium with 200 ng/mL aureobasidin (AbA) was used to confirm positive interactions (Figure S2). A sequencing analysis of the prey proteins from 100 randomly selected positive clones identified four TFs: a Nin-like family gene (*Vitvi02g00180*), a TALE family gene (*Vitvi08g00633*), a HD-ZIP family gene (*Vitvi08g01752*), and a ZF-HD family gene (*Vitvi18g00493*), all of which were expressed at the transcriptional level (Table S9, Figure S3). Notably, *Vitvi08g01752* was one of the predicted first-order regulators. The expression patterns of *Vitvi17g00329* (the second-order regulator), *Vitvi08g01752* (the first-order regulator), and *VvLOX3* were validated by quantitative real-time PCR (qRT-PCR) (Figure 7B–D). Those results suggest that the TO-GCN method can be used to identify the functional genes and upstream regulators of disease resistance in grapevine. Based on the regulatory network of *VvLOX3*, we proposed a simplified model for the resistance of GF to white rot in which *Vitvi17g00329* (L4), encoding an ERF family gene, regulates the expression of the *Vitvi08g01752* (L5), thereby enhancing the level of transcription of *VvLOX3*, which then produces more JA, and increases the resistance of GF to white rot (Figure 8). However, this model requires validation by additional study.

## 3. Discussion

Grapevine is one of the most valuable horticultural crops globally [22], but white rot significantly threatens its yield and quality. Therefore, understanding the mechanism of grapevine resistance to white rot is crucial for breeding resistant varieties. In this study, we observed significant differences in symptoms between GF and RG following infection with white rot. Noticeable lesions were apparent within 36 h in the RG variety, whereas the lesions on the GF variety were delayed until 36 h, and they were smaller and less severe than those on RG. This suggests that the GF variety suppressed the formation of lesions for a longer period of time after pathogen infection. We hypothesized that GF may initiate an effective defense mechanism at an early stage, thereby delaying the spread of pathogens. This is consistent with previous studies that indicated that plants can prevent the spread of pathogens through their early immune responses [23]. These phenotypic differences may stem from the distinct genetic backgrounds of the two grapevine varieties in their mechanisms of resistance to white rot. Previous research has shown that *V. labrusca* is generally highly resistant to diseases, particularly those caused by fungi, which is related to their evolutionary background and survival environment [24,25,26]. RG is a variety of *V. vinifera* L., while the GF variety, a hybrid of *V. vinifera* and *V. labrusca*, likely inherited its resistance genes from *V. labrusca*, which could have contributed to its strong resistance. These hypotheses require further validation through QTL mapping and comparative genomics approaches.

To elucidate the mechanisms that underlie the resistance of grapevine to white rot, we sequenced the transcriptome in the GF and RG varieties at seven time points after infection with the white rot pathogen. High-quality transcriptome sequencing data were used to identify a large number of expressed genes (16,461). Most of the expressed genes were shared between GF and RG, which indicated that these varieties might share similar basic defense mechanisms against white rot infection. Nevertheless, there were significant difference in the DEGs between GF and RG, particularly in the proportion of upregulated DEGs. GF had higher proportions across various time points compared to RG. Notably, there was a more active pattern of transcription in GF, and there were significantly higher numbers of upregulated genes at the key time points, particularly at 6 and 36 hpi. These results suggest that GF effectively activates a large number of defense-related genes during the early and middle stages of white rot infection, which enables the plants to activate their defense responses at an early stage. This active response in GF aligns with its phenotypic resistance characteristics and supports the crucial role of transcriptional regulation in plant disease resistance. In contrast, RG displayed a relatively weaker defense response, particularly during the early stages of infection, which may contribute to its susceptibility. The functional enrichment analysis of DEGs revealed that the core DEGs, rather than GF- or RG-specific DEGs, were significantly enriched in multiple GO terms related to plant disease resistance, such as responses to the ethylene, JA, and SA signaling pathways. These findings further suggest that GF can rapidly initiate defense mechanisms during the early stages of white rot infection, whereas RG fails to effectively activate sufficient defense genes.

TO-GCNs are an effective method to construct gene regulatory networks, which can calculate regulatory relationships between the TFs and various mechanisms of responses. Thus, this technique has been widely applied in plant research [17,18,19,27]. To analyze the temporal transcriptional regulation of GF and RG after infection with white rot pathogen, we constructed GF- and RG-specific TO-GCNs. Based on their patterns of expression, the levels of TO-GCN could be divided further into different stages (T0, T1, and T2). GF activated many TFs during the early infection stage (T1 stage), which may explain its initiation of an earlier defense response and stronger disease resistance. In contrast, the defense responses of RG were delayed, and there was only increased TF activity during the later stage (T2 stage). A functional analysis of the co-expressed genes in the specific-GCNs of both varieties revealed that GF enriched multiple GO terms related to plant disease resistance as early as the T1 stage (6–12 hpi), including such responses as wounding, oxidative stress, ethylene, and jasmonic acid. In contrast, RG at the same stage primarily enriched GO terms related to basic plant metabolism, such as photosynthesis and carbohydrate metabolism. These reactions indicate that RG may prioritize basic metabolic processes rather than specialized disease resistance responses during white rot infection. Additionally, during the T2 stage (24–72 hpi), the DEGs in GF continued to be enriched in multiple disease resistance-related terms, whereas those of RG were relatively limited and concentrated on the jasmonic acid response. This further supports the notion that GF can rapidly activate a broad range of defense responses early on, while RG responds more slowly and only activates a limited set of defense genes later in the infection process. This delayed response may be why RG is less effective at controlling the spread of the pathogen during the early stages of infection.

Plant hormones play a critical role in disease resistance, particularly ET, SA, and JA [28]. In this study, GF activated these hormones signaling pathways earlier and more significantly than RG, and the early activation of the JA signaling pathway was particularly prominent in GF (Figure 5). JA has been widely reported to be an important signaling molecule in plant disease resistance [5], and there were significantly higher levels of expression of the genes involved in the JA biosynthetic pathway in GF compared to RG. This provides additional evidence that JA may be one of the key reasons for the strong resistance to white rot in GF. Key enzymes in the JA biosynthetic pathway, such as LOX, AOS, AOC, and OPR, were significantly upregulated in GF following white rot infection, whereas their levels of expression in RG decreased or remained unchanged. The high levels of expression of these key enzymes facilitate the rapid production of JA, thereby enhancing the defense capabilities of GF. Notably, the LOX genes are present as tandem repeats on chromosome 6 (*Vitvi06g00149*, *Vitvi06g00150*, *Vitvi06g00153*, *Vitvi06g00155*, and *Vitvi06g00158)*, and the evolutionary development of this gene structure may have endowed GF with more resilience against pathogen invasion. Among these, *VvLOX3* (*Vitvi06g00158*) exhibited the highest transcriptional level and showed significant differences between GF and RG, and it is potentially one of the key factors that contributes to their differing disease resistance.

Many TFs play crucial regulatory roles in plant defense against pathogens [29]. However, there have been few studies on the regulators of *VvLOX3*. The use of a TO-GCN analysis in combination with TFBSs to identify regulators in the promoter region of *VvLOX3* led to the identification of several upstream regulators of *VvLOX3*, including members of the WRKY, NAC, and HD-ZIP families. WRKY proteins, as TFs that respond to stress, play important roles in plant immunity [6]. For example, in Arabidopsis, *WRKY53* can directly bind to the promoter regions of *LOX3/4*, thereby inhibiting the biosynthesis of JA and negatively regulating defense against herbivorous insects [30]. In this study, we found that several WRKY family members, including *WRKY6* (*Vitvi12g00388*), *WRKY7* (*Vitvi07g01694*), *WRKY22* (*Vitvi07g00421*), *WRKY47* (*Vitvi07g00523*), and *WRKY70* (*Vitvi13g01916*), may enhance the biosynthesis of JA by directly regulating the expression of *VvLOX3*, thereby increasing the resistance of GF to white rot. Previous research has shown that in *Nicotiana attenuata*, *NaWRKY6* may increase the levels of JA by regulating the expression of JA biosynthetic genes, such as LOX [31]. Additionally, we identified a NAC family TF, *Vitvi01g01038*, which is closely related to *NtNAC028* and *NtNAC080*. All three are homologous to *AtNAP* in *Arabidopsis*. Recent studies suggest that *NtNAC028* and *NtNAC080* can bind DNA on *NtLOX3*, but only the dimerization of the interactions between *NtNAC028* and *NtNAC080* has transcriptional activation activity [32]. We utilized Y1H assays to identify four TFs that can bind to the promoter region of *VvLOX3*, and *Vitvi08g01752* (a member of the HD-ZIP family) was also predicted to directly regulate *VvLOX3* by TO-GCN analysis. Recent studies have shown that in tomato (*Solanum lycopersicum* L.), an HD-ZIP family TF can directly regulate *SILOX*, thereby controlling the synthesis of hexanal and (Z)-2-heptenal in fatty acid metabolism [33]. These findings further suggest the importance of the HD-ZIP gene family in regulating the expression of LOX genes. Additionally, it is interesting to note that the other three genes, *Vitvi02g00180*, *Vitvi08g00633*, and *Vitvi18g00493*, all belong to the L4 level. Although the TO-GCN did not show their direct regulation of *VvLOX3*, their consistent presence at the level that immediately precedes *VvLOX3* (L5 and L6) suggests that their functions also merit further study.

Ultimately, we proposed a model: following white rot infection, the ERF family member *Vitvi17g00329* in GF regulates the HD-ZIP TF *Vitvi08g01752*, which, in turn, promotes the expression of *VvLOX3;* this enhances JA biosynthesis and grapevine resistance to white rot (Figure 8). This hypothesis provides a new perspective for further research on the mechanisms of resistance in GF. However, the exact mechanism that underlies this model requires further validation through functional experiments. For example, gene knockout or over-expression experiments could be used to determine the actual roles of these TFs in GF varieties, as well as their differential expression in resistant and susceptible varieties. Moreover, given the changes in these regulatory factors at different time points and levels of expression, further study into their dynamic regulatory mechanisms will provide more insights into the complexity of plant disease resistance.

## 4. Materials and Methods

### 4.1. Plant and Pathogen Materials

Two-year-old grapevine varieties, GF (resistant phenotype) and RG (susceptible phenotype), were cultivated in a greenhouse under controlled environmental conditions (25 °C, 80% relative humidity, 16 h light/8 h dark) at the Shandong Grape Research Institute, Jinan, China. The GF variety has American and European lineages, while the RG variety originated from Eurasia. The *C. vitis* pathogen strain GP1, stored in our laboratory, was cultured on solid potato dextrose agar (PDA) medium for 3 days at 28 °C. The disks of GP1 were used to evaluate the resistance of the GF and RG varieties. And the activated GP1 strain was also cultured in a liquid sporulation medium (1.5 g/L ammonium sulfate and 10 g/L grape branch sawdust) for 10 days at 28 °C with shaking at 180 rpm [34]. The spores were collected and diluted to a concentration of 1 × 10^6^ conidia/mL to infect the grapevine leaves. The GF and RG leaves were inoculated with the GP1 strain, and the samples were collected at 0, 6, 12, 24, 36, 48, and 72 hpi for RNA sequencing. There were three biological replicates for each experiment and six leaves per replicate.

### 4.2. RNA Extraction, Library Construction, and Sequencing

The total RNA was extracted using a CATB-pBIOZOL Kit (Bioflux, Redwood City, CA, USA). The concentration of RNA was measured using a NanoDrop 2000 (Thermo Fisher Scientific, Waltham, MA, USA), and the RNA integrity was assessed using an Agilent 2100 bioanalyzer (Agilent Technologies, Santa Clara, CA, USA). The extracted RNA was used to construct the cDNA libraries, and quality control was performed using the Agilent 2100 bioanalyzer. The cDNA libraries that were high-quality were sequenced on a DNBseq platform (MGI Tech, Shenzhen, China). The RNA-seq data have been deposited in the NCBI Short Read Archive database (SRA accession: PRJNA995417 and PRJNA1001063).

### 4.3. Transcriptome Analysis

The raw sequencing data were filtered using fastp v0.23.2 to remove reads with adapters [35], reads with <10% N content, or reads with >50% low-quality bases. The clean reads were aligned to the *V. vinifera* PN40024 12X.v2 reference genome using HISAT2 v2.2.1 [36,37]. The levels of gene expression were calculated as fragments per kilobase of transcription per Million mapped reads (FPKM) values using featureCounts v2.0.3 [38], and DEGs were analyzed with DESeq2 v1.22.1 with the *p*-values adjusted using the Benjamini and Hochberg method [39]. DEGs that were expressed in all the samples of either variety (FPKM > 1) were retained for further analysis. The R package clusterProfiler v4.6.0 was used for GO enrichment analyses and visualization of the DEGs [40].

### 4.4. TO-GCN

Time-ordered comparative transcriptome analyses were performed as described by a previous report [17]. Briefly, in this study, the input data consisted of two sets of time-series transcriptomes from the GF and RG grapevines following infection by the white rot pathogen. First, PCCs were calculated for 834 expressed TFs based on their FPKM values. All the PCC values were generated distributions of probability density function (PDF) and cumulative density function (CDF). According to the CDF, the positive and negative cutoff values were 0.85 and −0.78, respectively, with *p* < 0.05. Secondly, the focus was on the specific GCNs, namely GF+RG0 (GF-specific GCN) and GF0RG+ (RG-specific GCN). Third, the gene *Vitvi18g00973* was used as a seed to determine the temporal order of TF genes within each GCN using a breadth-first search (BFS) algorithm. For the TF genes at each level of a TO-GCN, a corresponding set of co-expressed genes (non-TF DEGs) was identified with the same co-expression relationship to add these genes to the TO-GCN.

### 4.5. qRT-PCR

For qRT-PCR, the total RNA was extracted using an RNA Purification Kit (TianGen, Beijing, China), and the RNA was reverse-transcribed using PrimeScript reverse transcriptase (TaKaRa, Dalian, China) according to the manufacturer’s instructions. Quantitative PCR was performed on a Roche Applied Science LightCycler 480 (Roche, Basel, Switzerland) using NovoStart^R^ SYBR qPCR SuperMix Plus (Novoprotein, Suzhou, China). The levels of relative mRNA expression were calculated using the 2^−ΔΔCt^ method [41], and grapevine *ACTIN7* (XM_002282480) was used as the internal reference gene. The specific primers used are listed in Table S10.

### 4.6. Y1H

Y1H assays were performed using the Matchmaker Gold Yeast One-Hybrid System (Takara Bio, Shiga, Japan). The 1998 bp *VvLOX3* promoter was amplified by PCR and inserted into the pABAi vector to generate pABAi-*VvLOX3*. The vector was linearized with *BstBI* endonuclease (NEB, Ipswich, MA, USA) and transfected into the Y1H Gold yeast strain to prepare competent cells. The Y1H library plasmid pGADT7, constructed with cDNA from grape leaves infected with *C. visit* and stored in our laboratory, was transformed into the competent cells. Transformants were selected and grown on SD/-Leu media with 100 ng/mL AbA to confirm the positive interactions. Positive prey proteins were amplified, sequenced, and analyzed by BLAST to determine the information of their genes.

## Data Availability

The original contributions presented in the study are included in the article and Supplementary Materials, further inquiries can be directed to the corresponding author.

## Supplementary Materials

The following supporting information can be downloaded at: https://www.mdpi.com/article/10.3390/ijms252111536/s1.
