# Supplementary material for: Time-Course Transcriptome Analysis Reveals Distinct Transcriptional Regulatory Networks in Resistant and Susceptible Grapevine Genotypes in Response to White Rot
Source: Int J Mol Sci. 2024 Oct 27;25(21):11536. doi: 10.3390/ijms252111536 (PMC11546955; doi:10.3390/ijms252111536)
Supplement: Supplementary file 1 [file ijms-25-11536-s001.zip › Supplementary Figures.pdf]

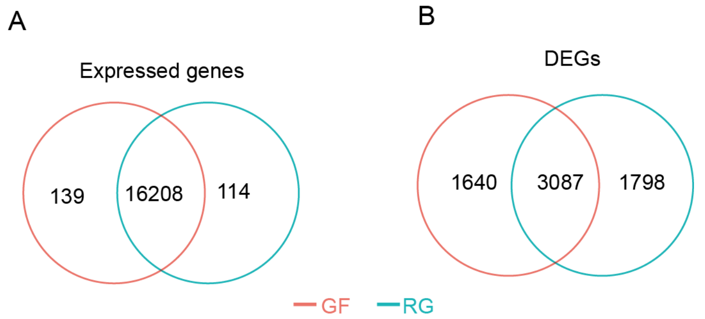

Figure S1 Comparison of gene expression patterns and functions between GF and RG varieties after inoculation with *C. vitis*. (A) Shared and unique expressed genes between GF and RG varieties. (B) Shared and unique DEGs between GF and RG varieties. The shared DEGs were categorized as core DEGs, while the unique DEGs were classified into RG-specific and GF-specific DEGs.

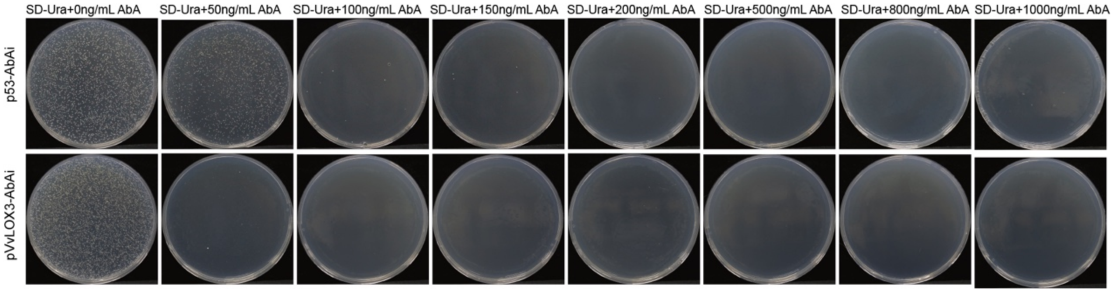

Figure S2 Determination of the Minimum AbA Inhibitory Concentration. A complete inhibition of strain growth was achieved at an AbA concentration of 100 ng/mL. Therefore, 100 ng/mL is the minimum inhibitory concentration of AbA for this experiment.

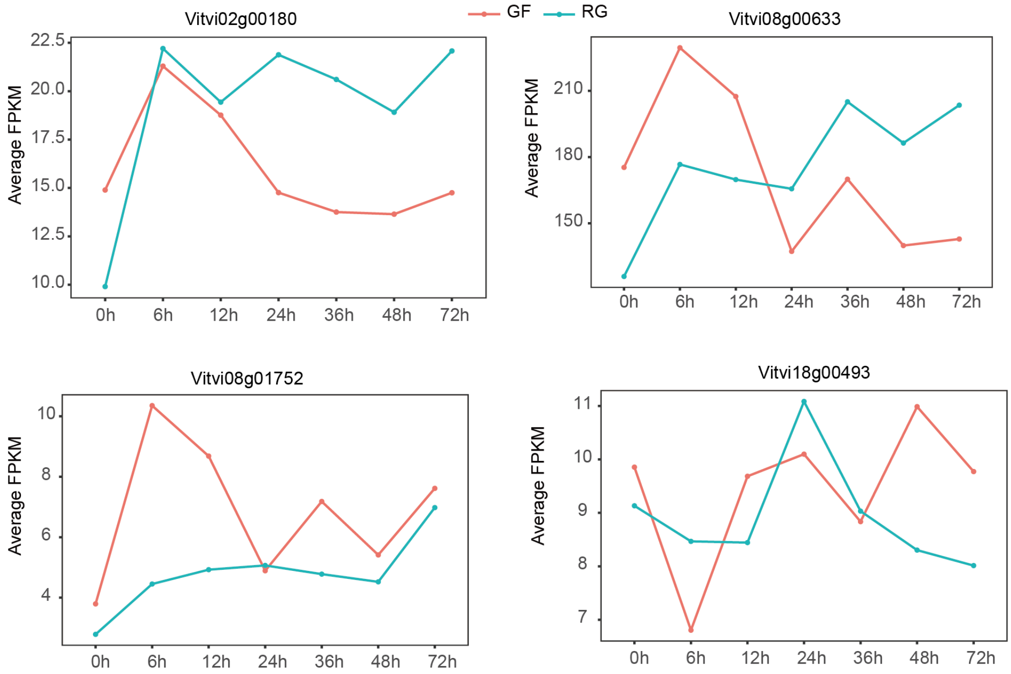

Figure S3 Expression patterns of four TF genes.
